# Configuration path analysis of Chinese government sports expenditure promoting national participation in physical activity

**DOI:** 10.3389/fpubh.2025.1538000

**Published:** 2025-03-04

**Authors:** Wenxin Zhu, Zhihao Du

**Affiliations:** School of Physical Education, China University of Mining and Technology, Xuzhou, Jiangsu, China

**Keywords:** government sports expenditure, physical activity participation, configurational pathway, fsQCA, fiscal expenditure

## Abstract

Focusing on the theoretical logic of Chinese government sports expenditure promoting public participation in sports, this study takes 31 provinces (cities, autonomous regions) in China as research objects and uses fsQCA to explore the configurational pathways of government sports expenditure affecting public participation in sports. Using the fuzzy set qualitative comparative analysis (fsQCA) method, and taking *the Sports Industry Statistical Yearbook (2020 data)* as the data source, this paper discusses how government sports expenditure can improve the level of public participation in sports through different combinations of driving factors. The results show that there are four effective condition combination paths, which can be summarized as the dual-core drive of “technological innovation + sports culture promotion and dissemination” and the “key housing support guarantee type” path, both of which are particularly crucial for improving public participation in physical activity. The findings of this study emphasize the importance of diversified investment in government sports expenditure for enhancing public participation in physical activity, suggesting that the government should adopt a cross-departmental resource integration strategy to build a comprehensive ecosystem supporting physical activity participation.

## 1 Introduction

### 1.1 The present situation of mass participation in physical activity

Physical activity participation pertains to the engagement of individuals in sports-related activities, either directly or indirectly. This involvement can be conscious or unconscious and is often influenced by personal emotional cognition and attitudes, leading to a series of related activities (1). The correlation between physical activity participation and individual physical health, social development, and the economic and social advancement of a country is well-established (2, 3). Research indicates that an increase in physical activity participation can lead to a reduction in healthcare costs (4) and an enhancement in labor productivity (5), thereby stimulating economic growth. Furthermore, physical activity participation holds significant social value as it fosters social interaction (6) and strengthens social cohesion (7), contributing positively to the construction of a harmonious society. However, as of the end of 2023, despite the fact that 37.2% of the Chinese population regularly engages in physical exercise (8), there remains a substantial gap from the target of “achieving a 38.5% proportion of regular physical exercise participants by 2025” as outlined in *the National Fitness Plan (2021–2025)* (9). The prevalence of mass physical activity participation in China is still lacking, necessitating further investment to boost public enthusiasm for sports. Consequently, the formulation of effective strategies to encourage mass physical activity participation and thereby elevate the national fitness development level is an urgent issue that requires immediate attention.

### 1.2 The importance and existing issues of government sports expenditure

The national government has demonstrated significant commitment to the development of a comprehensive fitness public service system, as evidenced by the issuance of documents such as *the National Fitness Program* and *Opinions on Building a Higher-Level National Fitness Public Service System* (10, 11). These initiatives underscore the government’s dedication to ensuring broad participation in sports activities. Fiscal expenditure serves as a crucial mechanism through which the government can facilitate the provision of public services and address societal needs. Specifically, government sports expenditure constitutes a vital component of overall fiscal spending, acting as an essential conduit for delivering sports-related public services and fulfilling the public’s athletic needs (12). This expenditure is pivotal in advancing national fitness objectives and enhancing the general health of the populace (13). However, there are notable challenges confronting China’s government sports fiscal expenditure at present. These include: an imbalance in the distribution of sports fiscal funds across various projects, inefficiencies in both the allocation and utilization of these funds, and a flawed internal structure of fiscal expenditure (14–16). Consequently, this results in a supply that fails to meet demand, culminating in a scenario characterized by both low efficiency and subpar quality (17). The persistence of these issues not only hampers the potential of sports investment to foster national fitness but also impedes the enhancement of governance efficiency within government sports public services. Resolving these challenges necessitates urgent policy adjustments and a strategic realignment of resource allocation.

Government sports fiscal expenditure empowers mass sports participation (18, 19). Sufficient financial investment can improve sports facilities, providing the public with convenient sports venues and lowering the barriers to participation. At the same time, funding supports the organization of sports events and activities, stimulating the public’s enthusiasm for sports and creating a positive sports atmosphere. Moreover, fiscal expenditure on sports education and training enhances the public’s sports skills and health awareness, further promoting the popularization and development of mass sports, forming a virtuous cycle, and driving the in-depth implementation of the national fitness strategy, improving the physical fitness and health level of the nation.

## 2 Research framework

### 2.1 Theoretical support

A strong correlation exists between governmental sports expenditure and public engagement in sports. Firstly, augmenting investment in sports enables the government to enhance sports infrastructure, offer additional sports-related public services, thereby fostering an environment conducive to public participation in sports (20–23). Conversely, a surge in public involvement in sports can stimulate the growth of the sports industry, boost the physical fitness of citizens, and consequently contribute to the nation’s economic development and societal stability (9).

From an “input-output” theoretical standpoint, government expenditure on sports significantly influences public participation in physical activities. As a pivotal component of public finance, such expenditure can bolster public engagement and awareness in sports by enhancing infrastructure, augmenting the availability of sports events and activities, and improving the quality of sports education and training (24). Government fiscal allocation toward sports is instrumental in advancing national fitness and elevating public health standards. Nevertheless, certain irrationalities in its expenditure structure, including inadequate investments in infrastructure development, educational outlays, and healthcare, constrain the potential of sports investment to foster national fitness. Consequently, policy refinements and resource optimization are imperative to refine the structure of government sports expenditure and augment fund utilization efficiency. This not only stimulates public fervor for sports but also enhances the quality and efficiency of sports public services. It addresses the escalating sports demands of the populace and underpins the high-quality evolution of sports public services. Such measures lay a robust groundwork for modernizing the national sports governance system and capabilities, act as a pivotal support for building a sports powerhouse, and form the cornerstone for effectively executing the national fitness strategy.

Public finance theory, which emphasizes the government’s pivotal role in the provision of public goods and services, is instrumental in understanding the allocation of government sports expenditure with sports public services being a significant representation of these functions (25). Sports public services are a critical component of these public goods, and the strategic deployment of government funds can significantly enhance the efficient use of resources and technology. This, in turn, improves the quality and accessibility of sports services, which are essential for fostering public participation in physical activities. New institutional economics provides a complementary perspective on the optimization of government sports expenditure structures. This theory posits that robust sports financial investment, as an institutional arrangement, is crucial for stimulating mass participation in physical activities (26). The structural optimization of government sports expenditure can reduce transaction costs and increase institutional efficiency, thereby promoting the effective distribution and utilization of sports resources (27). From the vantage point of new institutional economics, government sports expenditure is not solely focused on direct economic benefits but also takes into account the motivational impact of the institutional environment on public engagement in physical activities. This dual focus aims to achieve the maximization of social welfare by creating an environment that incentivizes and facilitates widespread participation in sports and physical activities.

### 2.2 Research objective

Government fiscal support invigorates the sports sector by encouraging the organization of sports events and promoting mass physical exercise, thereby stimulating residents’ enthusiasm for sports participation (28–30). The existing literature has accumulated a wealth of theoretical achievements on the academic study of national fitness and sports participation, laying a solid foundation for subsequent research. However, most studies focus on the micro-level aspects such as facility provision, with few exploring the characteristics from a macro-perspective and the micro-implications of fiscal policy. Specifically, issues concerning the optimization of sports finance to foster logical configuration of mass physical activity participation have not been systematically addressed. To address this gap, this study employs the fuzzy set qualitative comparative analysis (fsQCA) method to identify key configuration factors that influence physical activity participation through government sports expenditure. This will offer both a theoretical basis and practical guidance for the government in formulating sports policies and optimizing sports financial expenditure. This holds significant theoretical and practical implications for advancing the comprehensive development of national fitness participation and enhancing the health level of the population.

## 3 Research methods and data sources

### 3.1 Analytical framework

**Figure 1:**
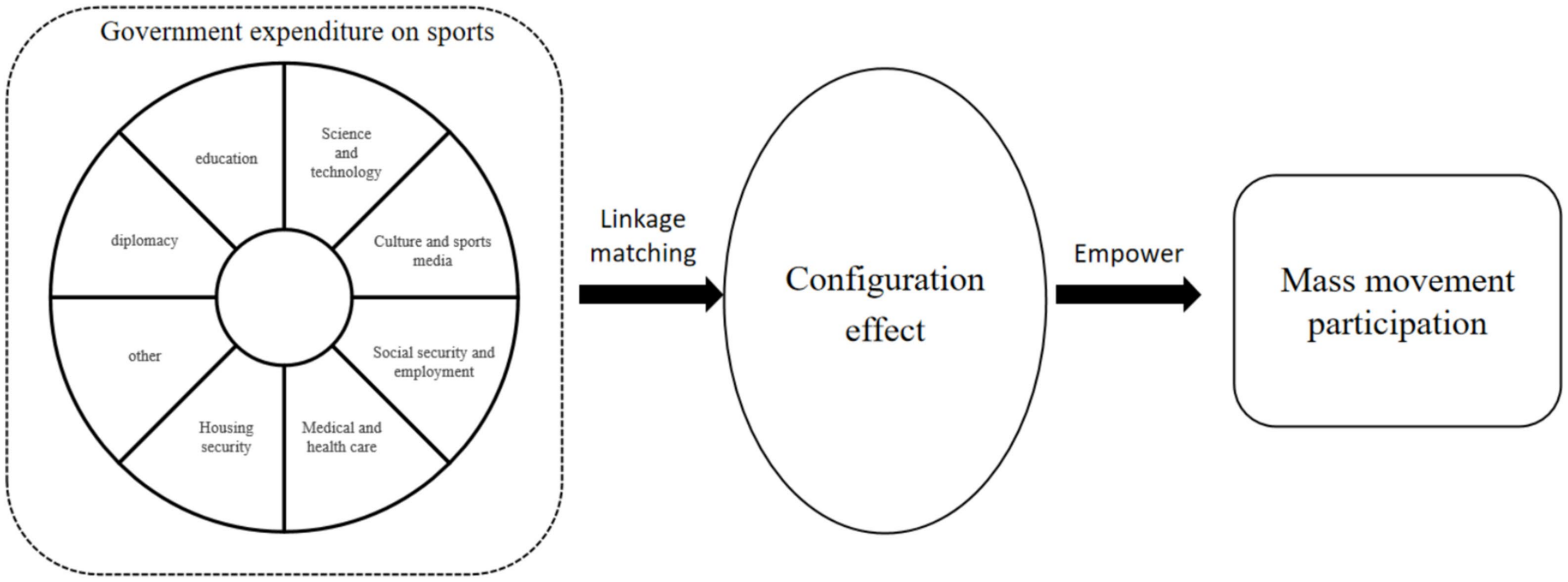
Analysis framework of government sports expenditure input to empower mass movement participation.

Government expenditure on sports encompasses various facets, each with distinct characteristics and potential impacts on mass sports. As outlined in *the Sports Industry Statistical Yearbook (2020 Data) (National System [2020] No. 12)*, sports fund revenue and expenditure items can be categorized into eight primary groups: diplomacy, education, science and technology, culture, sports and media, social security and employment, medical health, housing security, and miscellaneous. This categorization underscores the multifaceted roles and broad reach of government sports expenditure, with different financial contributions aligning with diverse aspects of basic public sports services (as shown in Figure 1).

① Diplomatic engagement plays a pivotal role in amplifying a nation’s international influence and competitiveness in the realm of sports, primarily through international sports exchanges and collaborations. For example, by hosting stages of the International Climbing Federation World Cup, both Keqiao District of Shaoxing City in Zhejiang Province and Wujiang District of Suzhou City in Jiangsu Province have elevated their global profiles and attracted increased investment for sports infrastructure. This has subsequently drawn more rock climbing enthusiasts and local residents to engage in sports activities. Consider the case of the 2024 Thomas and Uber Cup, set to be held in Chengdu. In preparation, the Chengdu municipal government has expanded the construction of badminton venues, thereby enhancing the comprehensiveness of sports facilities and boosting public participation in sports. Financial backing for sports diplomacy is instrumental in bolstering a nation’s international standing and competitiveness in sports, serving as a cornerstone for sports diplomacy and the shaping of international image.

② Investment in education is intrinsically linked to the development of sports talent and the dissemination of sports knowledge, forming the bedrock for enhancing national physical literacy. Such investment significantly influences public engagement in sports, serving not only to bolster the physical literacy of the youth but also to ignite their interest in sports through school-based activities and competitions (31). The funds allocated to physical education are channeled toward infrastructure development, teacher training, and the organization of sports activities, thereby providing students with ample opportunities to engage in physical exercise and cultivate consistent exercise habits. These investments enable students to derive pleasure from physical activity, improve their fitness levels, foster well-rounded personalities, and build resilience, ultimately leading to a higher rate of public participation in sports.

③ Investment in scientific and technological advancements fosters innovation in sports equipment and the application of science to sports training. This is achieved by supporting research activities within the sports domain, which in turn enhances sports performance and safety. The integration of advanced sports equipment, such as high-performance athletic shoes and smart fitness trackers, augments the safety and comfort of physical activities. These innovations not only mitigate the risk of sports-related injuries but also facilitate real-time monitoring of athletic data, thereby encouraging broader public engagement in sports (32). For instance, the advent of intelligent wearable technology offers users tailored fitness insights and regimens, thereby amplifying both the enjoyment and challenge of sports. Furthermore, strides in sports science and technology have ushered in evidence-based training methodologies, such as exercise prescriptions grounded in sports physiology research. These methods augment the efficacy and professionalism of sports while promoting widespread participation. Regulatory documents like the “Extracurricular Sports Training Behavior Norms” issued by the General Administration of Sport of China serve to standardize youth sports training, elevate its quality, and cater to the training requirements of young individuals (33). Such investments and regulations not only amplify the scientific rigor and appeal of sports but also bolster the growth of sports education. Consequently, this provides the populace with an array of sports options and accessible conditions, thereby boosting public engagement in sports.

④ Investments in cultural, sports, and media encompass contributions to administrative operations, overarching administrative management, institutional services, project management within sports, competitions, training, venues, mass sports initiatives, exchanges and collaborations in sports, along with other expenditures categorized under cultural media. Such investments are pivotal in molding the sports culture and amplifying public engagement with sports, thereby epitomizing the soft power of culture.

⑤ Investment in social security and employment within the sports industry signifies the government’s commitment to providing social security for practitioners, as well as offering financial support to encourage employment through sports activities. This plays a crucial role in maintaining stability within the sports industry and fostering social harmony. Such investment promotes widespread participation in sports by facilitating job training and supporting entrepreneurship (34). Government funding aids in the training of sports professionals, such as fitness coaches, thereby ensuring the provision of high-quality sports services. This, in turn, stimulates enthusiasm for mass sports, such as the transformation of old facilities into sports consumption venues (35). These initiatives create convenient sports conditions for various demographic groups, including young people, middle-aged and older adult individuals, and enterprise employees (36). They also foster an interest and habit of sports, thereby enhancing the participation of the general public in sports.

⑥ Investment in healthcare for athletes emphasizes medical protection and hygiene safety during sports activities, thereby ensuring the healthy progression of these events. Such investment offers medical rehabilitation support and preventive healthcare services, providing a safety net for public participation in sports and encouraging widespread involvement in physical activities (37). The dissemination of sports health knowledge has heightened public awareness regarding the prevention of sports injuries. The implementation of scientific physical monitoring and health education initiatives has bolstered the efficacy of fitness programs. Furthermore, the introduction of sports insurance services has mitigated concerns about sports-related risks, collectively amplifying public enthusiasm for participation in sports.

⑦ Investment in housing security, which encompasses both athlete accommodation and land tenure for sports facility construction, is pivotal for retaining sports talents and enhancing the quality of sports infrastructure. Such investments bolster sports facilities by funding community sports infrastructure and allocating governmental special funds, thereby offering accessible exercise venues for the populace (38). Notably, the enhancement of community sports facilities has significantly benefited various demographics, including the older adult, youth, and families (39). The older adult benefit from a secure exercising environment, while young individuals gain diverse opportunities for physical activity and social interaction. Families, on the other hand, find these facilities conducive to strengthening parent–child bonds. The accessibility of these amenities encourages the cultivation of regular exercise routines across all age brackets, leading to a surge in overall physical activity participation rates.

⑧ Other inputs encompass sports-related expenditures that cannot be explicitly categorized into the aforementioned categories, thereby providing flexible financial support for the comprehensive development of sports. These additional sports inputs enhance public awareness of sports by funding public welfare activities and regional sports events. They also offer a variety of sports activities for different regions, collectively promoting the development of national fitness (40). These inputs serve as a supplement and emergency response capability for sports development, and are a crucial guarantee for its diversified growth.

### 3.2 Research methods and variable measurement

#### 3.2.1 Qualitative comparative analysis

This study employs Qualitative Comparative Analysis (QCA) as its primary research methodology, a technique introduced by Charles Ragin in 1987 and grounded in set theory and Boolean logic (41). QCA is an approach that bridges qualitative and quantitative research, offering the ability to discern various factor combination pathways (42). It is particularly adept at handling small to medium-sized datasets, revealing intricate causal links between diverse conditional factor configurations and specific outcomes (43). Within QCA, there are crisp set strategies, fuzzy set strategies, and multi-valued set strategies. Given the nuanced nature of factors influencing public due to government sports expenditure, it’s impractical to categorize either the conditional or outcome variables with a straightforward binary “yes” or “no.” The inherent ambiguity in the degree and level of these variables suggests a significant potential for information loss. Consequently, this study opts for the fuzzy set strategy within QCA (24).

Given the complexity of the system involving multiple interacting factors in this study, fuzzy-set qualitative comparative analysis (fsQCA) is well-suited to examine the impact of government sports expenditure on mass physical activity participation. Firstly, the relationship between these two variables is inherently complex, encompassing numerous interdependent factors. Unlike traditional regression methods, fsQCA is better equipped to handle non-linear relationships within complex systems, elucidating systematic characteristics and asymmetries resulting from element interactions. This is crucial for understanding the multifaceted ways in which government sports expenditure influences mass physical activity participation. Secondly, the factors influencing the impact of government sports expenditure on mass physical activity participation exhibit a degree of fuzziness that traditional quantitative methods fail to accurately capture. By employing fuzzy set data, fsQCA can more precisely delineate the intricate relationships between variables, thereby uncovering potential mechanisms and diverse pathways leading to varying levels of physical activity participation. Thirdly, fsQCA underscores the concept of “equivalent causal chains,” suggesting that different combinations of conditions can yield identical outcomes. This allows for a more comprehensive exploration of the diversity and complexity inherent in the ways government sports expenditure facilitates mass physical activity participation, offering a broader perspective for the study.

### 3.3 Variable measurement

#### 3.3.1 Sample selection

In adhering to the normative standards of QCA modeling, the selection of samples for fuzzy-set qualitative comparative analysis (fsQCA) must adhere to the following criteria: Firstly, sample homogeneity should be ensured, meaning that factors external to the outcome and condition variables ought to be largely consistent to prevent any undue influence on the model’s outcomes. Secondly, it is imperative to guarantee sample diversity, encompassing various categories of case samples (44). Thirdly, sample comparability is crucial; selected case samples must exhibit consistency or a high degree of similarity in defining outcome and condition variables, as well as in their measurement methodologies. Lastly, sample sufficiency must be observed, ensuring that the sample size is adequately large to encompass all potential configurations of condition variables, thereby mitigating the risk of logical remainders (45).

In light of the stipulated criteria and taking into account data availability, completeness, and comparability, this research incorporates 31 provinces (autonomous regions, municipalities) in China as its sample base, employing provincial-level data for configuration analysis (refer to Table 1). The year 2020 is designated as the data benchmark, representing a pivotal juncture when China achieved comprehensive construction of a moderately prosperous society and initiated its second centenary goal. This year also marked the culmination of *the National Fitness Plan (2016–2020)* (46) and the strategic layout for the *14th Five-Year Plan for Sports Development* (47). In 2020, national fitness, recognized as a national strategy, underscored its multifaceted functions and values, with its popularization serving as a significant barometer of the nation’s modernization trajectory. Consequently, this study leverages pertinent data from government sports expenditures in 2020 to bolster mass physical activity participation as the analytical sample, thereby ensuring data timeliness and judicious contemplation of potential lagging effects from various determinants.

**Table 1:** Provinces from which samples were selected for this study.

Number	Province
1	Beijing
2	Tianjin
3	Hebei
4	Shanxi
5	Neimenggu
6	Liaoning
7	Jilin
8	Heilongjiang
9	Shanghai
10	Jiangsu
11	Zhejiang
12	Anhui
13	Fujian
14	Jiangxi
15	Shandong
16	Henan
17	Hubei
18	Hunan
19	Guangdong
20	Guangxi
21	Hainan
22	Chongqing
23	Sichuan
24	Guizhou
25	Yunnan
26	Xizang
27	Shaanxi
28	Gansu
29	Qinghai
30	Ningxia
31	Xinjiang

### 3.4 Indicator system

#### 3.4.1 Measurement of outcome variables

The primary focus of this study is the extent of mass physical activity participation, thus the rate of such participation in each province is considered as the outcome variable for physical activity participation level (refer to Table 2). This research employs the proportion of individuals who regularly engage in physical activities relative to the permanent population of each of China’s 31 provinces (including autonomous regions and municipalities) in 2020. This ratio serves as the measurement indicator for the degree of mass physical activity participation within a given region. The data utilized in this study is sourced from the *14th Five-Year Plan for Sports Development* (47) and the *National Fitness Implementation Plan (2021–2025)* (48) released by each province, supplemented by relevant government work reports.

**Table 2:** Description of variable indicators and data sources.

	Variable	Describe indicator	Data sources
Outcome variable	Physical activity participation rate	The ratio of the number of people who frequently participate in sports in each province in 2020 to the permanent population of that province in 2020. (Data unit: %).	*The 14th Five-Year Sports Development Plan*, *the National Fitness Implementation Plan (2021–2025)*, and related government work reports released by various provinces.
	Diplomatic Investment	The amount invested by provincial government departments in sports diplomacy in 2020. (Data unit: RMB 10,000)	*Statistical Yearbook of Sports Industry (Data for 2020)*
	Educational Investment	The amount invested by provincial government departments in sports education in 2020. (Data unit: RMB 10,000)	
	Scientific and Technological Investment	The amount invested by provincial government departments in sports science and technology in 2020. (Data unit: RMB 10,000)	
Condition variable	Culture and Sports Media Investment	The amount invested by provincial government departments in sports under the culture and sports media investment in 2020. (Data unit: RMB 10,000)	
	Social Security and Employment Investment	The amount invested by provincial government departments in sports social security and employment in 2020. (Data unit: RMB 10,000)	
	Healthcare Investment	The amount invested by provincial government departments in sports healthcare in 2020. (Data unit: RMB 10,000)	
	Housing Security Investment	The amount invested by provincial government departments in sports housing security expenditures in 2020. (Data unit: RMB 10,000)	
	Other Investments	The amount invested by provincial government departments in other sports-related endeavors in 2020. (Data unit: RMB 10,000)	

#### 3.4.2 Conditional variable measurement

The *Sports Industry Statistical Yearbook* stands as the foremost authoritative statistical publication on sports in China. It offers a comprehensive overview of the nation’s sports development and is primarily derived from the annual sports industry statistics reports of every province, autonomous region, and municipality under direct central government administration. Currently, the Sports Economic Department of the General Administration of Sport of China produces this volume annually (49).

The typological capability of fsQCA (42), in conjunction with *the Sports Industry Statistical Yearbook (2020 Data) (National Statistics [2020] No. 12)*, allows for the establishment of conditional variables based on the categorization of sports funding revenue and expenditure. These categories encompass diplomacy, education, science and technology, culture, sports and media, social security and employment, medical health, housing security expenditure, among others, resulting in a total of eight conditional variables (refer to Table 2). The *Sports Industry Statistical Yearbook* stands as the most authoritative official statistical resource on sports in China, offering the sole comprehensive insight into the development of national sports. The data primarily originates from the annual sports industry statistics reports, which are compiled by the sports commissions of each province, autonomous region, and municipality directly under the central government. Currently, a volume is produced annually by the Sports Economic Department of the General Administration of Sport of China.

① Diplomatic Investment in Sports. The allocation of resources toward sports diplomacy fosters international collaboration, facilitates the introduction of cutting-edge facilities and technologies, and augments the development of domestic sports infrastructure. This, in turn, amplifies the rate of participation in mass sports (50). Governmental investment in this domain encompasses financial backing for international sports interactions and collaborations, which includes expenses related to bidding for global events and membership dues for international sports associations.

② Investment in Education. The investment in sports education encompasses both general and vocational education, with a particular emphasis on the allocation of funds for sports-related instruction and training. This investment is intrinsically linked to the development of sports talent and the dissemination of sports knowledge.

③ Investment in Science and Technology. This encompasses the allocation of resources toward scientific and technological advancements in sports, including applied research and technical development. It involves the application of scientific principles to sports training and the innovation of sports equipment.

④ Investment in Cultural, Sports, and Media. This form of investment encompasses the promotion of sports culture and the distribution of sports events, which includes acquiring broadcasting rights for such events and fostering sports culture to stimulate public interest (51). Specifically, it involves expenditures related to administrative operations, general administrative management, institutional services, sports project management, competitions, training, venues, mass sports, exchanges and cooperation, as well as other sports-related expenses within the cultural media sector.

⑤ Investment in Social Security and Employment. This category represents the government’s commitment to providing social security for professionals within the sports industry, as well as retirees. It also encompasses financial incentives aimed at fostering employment opportunities through sports-related activities.

⑥ Healthcare Investment. The allocation of resources toward sports healthcare primarily emphasizes the medical safeguarding of athletes and the maintenance of hygiene standards in sporting events, thereby ensuring the sustainable development of such activities.

⑦ Investment in Housing Security. The financial commitment to sports-related housing security primarily encompasses expenditures associated with housing reform. This includes the provision of housing support for athletes and economically disadvantaged groups, as well as ensuring land security during the construction of sports facilities.

⑧ Miscellaneous Expenditures. This category encompasses sports-related expenditures that do not fit neatly into the aforementioned categories, thereby offering flexible financial support for the holistic development of sports.

### 3.5 Data calibration

The calibration procedure for fuzzy set qualitative comparative analysis necessitates the assignment of membership degrees, ranging from 0 to 1, to the cases under investigation. The primary objective of this process is to ascertain the precise position and significance of these values, essentially interpreting their degree of membership to either high or low score sets. In this study, we employ the conventional direct calibration method, establishing three anchor points: “complete membership,” “crossover point,” and “complete non-membership.” These are determined by scrutinizing the actual value distribution of variables across the cases. Guided by extant research practices and the inherent data characteristics of our cases, we use the 95, 50, and 5% quantiles of the sample data as anchor points for both the outcome variable—mass movement participation rate—and the eight conditional variables: government sports expenditure in diplomacy, education, science and technology, culture and sports media, social security and employment, medical health, housing security, among others. Specifically, these anchor points are denoted as (0.95, 0.5, 0.05) (52), ensuring both the precision of calibration and the reliability of the ensuing results. Consequently, fsQCA3.0 software is utilized to calibrate these nine variables, transforming them into fuzzy set membership variables within a 0–1 range. The specific settings for these calibration anchor points are detailed in Table 3.

**Table 3:** Calibration of variables.

	Variable	Complete membership 95%	Crossover point 50%	Complete non-membership5%
Outcome variable	Physical activity participation rate	0.49	0.39	0.30
	Diplomatic investment	125.18	0.00	0.00
	Educational investment	25143926.94	22478.58	169.48
	Scientific and technological investment	5382.41	72.06	0.00
Condition variable	Culture and sports media investment	367357.88	112637.69	36099.59
	Social security and employment investment	16791.62	2326.00	190.54
	Healthcare investment	6925.70	1732.10	236.80
	Housing security investment	24352.58	2291.51	161.37
	Other investments	242169.07	21795.70	2813.88

## 4 Research results and analysis

### 4.1 Univariate conditional necessity test

Prior to conducting the configuration analysis, it is imperative to perform a necessity analysis on the conditional variables. This is done to ascertain whether a single factor acts as a necessary condition for the outcome variable (53). Initially, this study carried out a necessity test on the eight conditional variables of government sports expenditure. The aim was to determine if there exists a single factor that has a decisive impact on mass physical activity participation (the results are presented in Table 4). The analysis of the data revealed that none of the consistency values of all conditions surpassed the critical value of 0.90. This suggests that no single condition can be deemed as a necessary condition for explaining its result under the high or low level of mass physical activity participation.

**Table 4:** Univariate necessary condition analysis.

Conditional variables	High participation level		Low participation level	
	Consistency	Coverage	Consistency	Coverage
High Diplomatic Investment	0.786	0.716	0.779	0.779
Low Diplomatic Investment	0.757	0.757	0.716	0.786
High Education Investment	0.591	0.795	0.574	0.847
Low Education Investment	0.886	0.655	0.861	0.698
High Science and Technology Investment	0.569	0.73	0.522	0.728
Low Science and Technology Investment	0.786	0.6	0.801	0.671
High Culture and Sports Media Investment	0.737	0.701	0.655	0.684
Low Culture and Sports Media Investment	0.667	0.638	0.713	0.749
High Social Security and Employment Investment	0.610	0.688	0.586	0.725
Low Social Security and Employment Investment	0.756	0.625	0.747	0.678
High Medical and Health Care Investment	0.654	0.727	0.609	0.742
Low Medical and Health Care Investment	0.768	0.642	0.609	0.742
High Housing Security Expenditure Investment	0.627	0.720	0.587	0.740
Low Housing Security Expenditure Investment	0.774	0.630	0.779	0.696
High Other Investments	0.664	0.712	0.638	0.752
Low Other Investments	0.769	0.659	0.756	0.712

The findings underscore the intricate nature of the factors influencing the impact of government sports expenditure on mass physical activity participation at the provincial level. This suggests that a combination of conditional variables is essential to foster elevated levels of mass physical activity participation through their collaborative interaction and alignment. Consequently, various dimensions of government sports expenditure—including diplomacy, education, science and technology, culture and sports media, social security and employment, medical health, housing security, among others—need to collectively activate the motivational mechanisms for mass physical activity participation by leveraging their combined effects.

### 4.2 Conditional configuration sufficiency analysis—fsQCA

This study employs the fsQCA method to perform a truth table analysis, scrutinizing the sufficiency of government sports expenditure configurations across various provinces in relation to high and non-high physical activity participation levels. To ensure precise case distribution and establish an appropriate frequency threshold based on sample size, the minimum case frequency is set at 1 and the threshold is established at 0.8. The standard rows with PRI values less than 0.65 underwent manual zeroing processing (54). Using the fsQCA 3.0 software, multiple paths were computed, yielding three distinct solutions: simple, intermediate, and complex. Given the limited adaptability of both the simple and complex solutions, this study opted for the simple solution as a reference point. The intermediate solution was selected as the final path outcome. This research identified four composite scheme conditions for government sports expenditure to bolster mass physical activity participation (55). The core and edge conditions for each configuration were then delineated, and metrics such as consistency, original coverage rate, and other pertinent characteristic values for both individual and overall solutions were meticulously detailed (24) (refer to Table 5).

**Table 5:** Configuration of conditions for enabling mass physical activity participation by government sports expenditure.

Conditional item	Path ①	Path ②	Path ③	Path ④
Diplomatic Investment	·	·	·	·
Educational Investment		·	⊗	·
Scientific and Technological Investment	●	●	·	·
Culture and sports media Investment	●	●	·	·
Social security and employment Investment	·	⊗	⊗	⊗
Healthcare Investment	⊗	⊗	·	·
Housing security Investment	·	⊗	●	●
Other Investments	·	●	⊗	·
Consistency	0.943203	0.942514	0.943735	0.952393
Raw Coverage	0.337077	0.255142	0.295061	0.257172
Unique Coverage	0.067118	0.021042	0.036604	0.008863
Consistency of Solution	0.934799			
Coverage of Solution	0.417118			

● indicates the presence of a core condition; · indicates the presence of a marginal condition; ⊗ indicates the absence of a core condition; ⊗ indicates that this marginal condition does not exist, and “blank” means that this condition can either exist or not exist.

The analysis results reveal that, based on the actual cases in 2020, there are four configurations for government sports expenditure that can enhance mass physical activity participation (see Table 5). Path ① has a coverage rate of approximately 33.71%, accounting for about 33.71% of the high physical activity participation level cases; Path ② has a coverage rate of around 25.51%, explaining roughly 25.51% of the high physical activity participation level cases; Path ③ has a coverage rate of about 29.50%, accounting for approximately 29.50% of the high physical activity participation level cases; and Path ④ has a coverage rate of approximately 25.72%, explaining roughly 25.72% of the high physical activity participation level cases. The consistency levels of both individual solutions and overall solutions for government sports expenditure to boost mass physical activity participation are relatively high, at 0.942514 and 0.952393, respectively. This indicates that the identified four condition combinations have a high degree of internal consistency, with an overall consistency of 0.934799. This suggests that among the cases conforming to these four configurations, about 93.48% have higher project governance efficiency. The total coverage rate reaches 0.417118, indicating that the four configurations can cover about 41.71% of the cases where government sports expenditure enables a higher level of mass physical activity participation. This provides robust empirical support for how government sports expenditure can effectively promote mass physical activity participation.

### 4.3 Robustness test

The current robustness tests for QCA encompass: the adjustment of the consistency threshold and the elimination of certain samples (56), alteration of the measurement method (57), and reverse testing of outcome variables (58). This study employs the technique of adjusting the consistency threshold for robustness testing, setting the consistency level to 0.85. The configuration path obtained post-adjustment aligns with the promotion path identified in the original analysis, thereby further corroborating the stability of the research findings.

## 5 Influence path and analysis

Adhering to the principles for naming configurational solutions as outlined by Furnari et al. (59), which emphasize “concise expression,” “capturing the whole,” and “evoking the essence of the configuration,” this article strives to ensure the rationality of the naming while considering the integrity and uniqueness of the configurations. In line with these principles and to provide clearer and actionable implementation pathways for enterprises, this study refrains from individually naming each configuration. Instead, it categorizes configurations based on the principle of capturing overall patterns (60). Consequently, configurations with identical core conditions from the analysis results in Table 5 are consolidated into two patterns: the “Dual-Drive Model of Science and Technology Innovation and Sports Culture Promotion and Communication” and the “Key Housing Support and Security Model.” These patterns are explored to understand how government sports expenditure empowers mass sports participation.

The discussion section of this article will focus on these two core pathways. Initially, the “Dual-Drive Model of Science and Technology Innovation and Sports Culture Promotion and Communication” will be explored, highlighting the pivotal role of science and technology as well as media and culture in enhancing mass sports participation. Subsequently, the “Key Housing Support and Security Model” will be examined, analyzing how housing security expenditure can improve residents’ living environments and sports conditions, thereby further promoting sports participation. The analysis of these pathways aims to provide theoretical support for the optimized allocation of government sports expenditure, facilitate the effective utilization of resources, and advance the development of national fitness goals to a higher level.

### 5.1 “Technology innovation + sports culture promotion and dissemination” dual-core driven model

The explanatory power of both Configuration Path ① and Configuration Path ② is robust, with their consistency rates being approximately 93.32 and 94.25%, respectively. The findings from the configuration path analysis suggest that when scientific and technological investment, along with cultural and sports media investment, are considered core conditions—and are bolstered by auxiliary conditions such as diplomatic investment—government sports expenditure can significantly enhance mass participation in sports. When scientific and technological investment and cultural and sports media investment are viewed as core conditions, Configuration Path ① furthers high levels of mass physical activity participation through investments in diplomacy, social security, employment, housing security, among other sectors. Conversely, Configuration Path ②, while considering scientific and technological investment, cultural and sports media investment, and other investments as core conditions, facilitates high levels of mass physical activity participation via diplomatic and educational investments. In the dual-core driven configuration mode of “technological innovation + sports culture promotion and dissemination,” the sports expenditure strategies of the Beijing, Jiangsu, and Hebei governments in 2020 serve as prime examples of elevating mass physical activity participation in their respective regions. Taking Beijing as an example, in terms of investment in sports science and technology, the Beijing Municipal Government supports scientific research activities in the field of sports, promotes innovation in sports equipment and scientification of sports training, thereby improving sports performance and safety. In 2020, the Beijing Municipal Bureau of Sports underscored the integration of technology in sports. This included exploring advanced technological applications such as “blockchain,” “5G,” “8 K,” and “VR.” These technologies not only elevated the professionalism of sports training and competitions but also piqued public interest and participation in emerging sports activities (61). The Beijing municipal government has played a pivotal role in fostering a positive sports culture by endorsing sports culture promotion, event management, and dissemination. Furthermore, Beijing has been proactive in organizing a diverse range of national fitness competitions and sports events. Districts have cultivated branded sports events, adopting themes like “one district, one product,” “one street (township), one product,” and “one community (village), one product.” This has led to a dynamic scenario where communities engage in daily activities, monthly competitions, and annual sports meetings. Leveraging the potential of “Internet+,” Beijing has spearheaded an innovative operational model for the Olympic City Sports Culture Festival. This festival, based in Beijing, extends its reach nationwide and globally, further propelling the legacy of the Olympics and bolstering urban development (62).

The government’s investment in technological innovation within the sports sector has catalyzed advancements in this field. As scholars such as Huang Qian have posited, technological innovation plays a pivotal role in enhancing public participation in sports, offering the masses scientific, efficient, and intelligent methods of engagement, as well as comprehensive health management solutions (63). Echoing this sentiment, Srikanth and Srikanth (64) also asserts that technological innovation in sports can boost participation levels. Key technological advancements, including wearable devices, fitness applications, virtual and augmented reality, performance analysis tools, and smart sports equipment, are instrumental in increasing engagement among students and athletes (64). Technological innovation offers novel platforms and tools for enhancing the promotion and dissemination of sports culture. Through data analysis and intelligent devices, it enables more precise and personalized delivery of sports information, individualized recording of physical activity participation, and the development of smarter sports venues (65). Concurrently, as discovered by scholars such as Huang Zhuo, investments in culture and sports media can significantly enhance public participation in sports (66). In this process, the advancement of sports infrastructure is particularly crucial, providing a solid foundation for the development of mass sports. Scholars like Wang Zhihui further point out that by strengthening infrastructure, the popularization and development of mass sports can be effectively promoted, thereby improving the health levels and quality of life of the public (67). Investments in culture and sports media bolster administrative operations, general management, institutional services, sports project oversight, competitions, training, venue management, mass sports initiatives, and international sports collaborations. This is achieved by refining sports supply services, advancing facility construction, and elevating training standards. In this context, technological innovation acts as both a technical conduit and a medium for fostering a sports culture ambiance, subtly nurturing public interest and engagement in sports, leading to a collective impact. The interplay between technological innovation and sports culture promotion results in a mutually reinforcing synergy. By elevating the intelligence and digitalization of sports infrastructure, technological innovation ensures a more judicious and efficient distribution of sports resources, thereby enhancing accessibility and participation in sports activities.

### 5.2 Key housing support security type

According to Table 3, the consistency of Configuration Path ③ and Configuration Path ④ reached 94.37 and 95.23%, respectively, with Configuration Path ④ demonstrating the strongest explanatory power among the four paths. Configuration Paths ③ and ④ indicate that, against the backdrop of inadequate social security and employment investment, the government can effectively enhance the level of public physical activity participation by increasing expenditure on housing security. The practices of the Shanghai and Guangxi governments in 2020 serve as a typical example of this empowering process, successfully stimulating the public’s enthusiasm for physical activity participation through government expenditure on sports housing security. Taking Shanghai as an example, the government has paid attention to and supported the housing security of practitioners in the sports field. The Shanghai Sports Bureau allocated 30.3 million yuan in the 2020 budget for expenses such as the payment of housing provident funds for on-duty personnel. This financial investment not only improved the welfare of sports industry practitioners but also indirectly promoted the construction and maintenance of sports facilities, providing a better sports environment for the public.

In the “14th Five-Year Plan,” the Shanghai Sports Bureau proposed to achieve full coverage of urban sports centers at the district level, and essentially cover all streets and towns with community fitness centers and citizen fitness stations (68). This means that by supporting the construction and improvement of community sports facilities, convenient sports venues are provided for residents, enabling them to easily participate in sports activities within their communities, thereby enhancing their enthusiasm for sports. By the end of 2020, there were a total of 19 district-level sports centers in the city, essentially achieving a balanced layout of high-grade sports facilities. The Citizen Sports Park (Phase I) Soccer Park has been completed and opened, the Pudong Football Stadium is essentially completed, the Xujiahui Sports Park and the Jiushi International Equestrian Center projects have commenced construction, and the development of sports facilities for major events and public sports facilities continues to advance. Shanghai has also strengthened the integrated development of sports facilities through investment in sports housing security, adopting models such as “Sports + Greening,” “Sports + Transportation,” and “Sports + Education,” making full use of urban space resources, increasing the supply of fitness facilities, and improving the efficiency and quality of sports facilities (69). The completion of these projects has not only increased the quantity and quality of sports facilities but also enhanced the public’s participation and satisfaction with sports activities.

Increasing investment in housing security can effectively promote public participation in sports. Fiscal expenditures on housing security are capable of enhancing the basic living conditions and housing circumstances of the public. Li Xiaotian and the German sociologist Karl Mannheim have both noted that individuals engaging in fitness activities exhibit a stratified characteristic, with family income, housing ownership, housing size, and education serving as elements for social class differentiation (70–72). According to scholar Zheng Jiakun, under the premise of increased fiscal investment aimed at improving the basic living and housing conditions of the public, the development of mass sports can be effectively advanced (73). The increase in housing security expenditure provides a more stable living environment for low-income families, thereby reducing life pressures and increasing the time and energy available for these individuals to engage in sports activities (74). The implementation of housing security policies is often accompanied by the construction and improvement of community sports facilities (75). While providing housing support, governments often concurrently build sports venues and public sports facilities, the convenience and accessibility of which directly promote public physical activity participation. The construction and improvement of sports venues provide a foundational guarantee for the development of mass sports, offering venue support to increase the willingness and ability to participate in sports (76).

## 6 Conclusion and implications

### 6.1 Research conclusion

The study discerned four distinct governance pathways through which governmental sports expenditures bolster mass participation in sports, categorized into two primary levels. The first level pertains to the “technological innovation + sports culture promotion and dissemination” dual-core driven path, wherein investments in scientific and technological advancements, as well as cultural and sports media, serve as the pivotal conditions. Conversely, the second level is characterized by the “key housing support guarantee type” path, where investment in housing security emerges as the principal driving factor.

The examination of the prerequisite conditions for the QCA method reveals that no single condition variable is indispensable for facilitating government sports expenditure to augment mass physical activity participation. Furthermore, no individual condition variable can independently induce such an outcome. In other words, the influence of a solitary governance element on empowering mass physical activity participation through government sports expenditure is constrained. Consequently, to enhance governance efficiency, it is imperative to prioritize core conditions and amalgamate them with supplementary conditions to harness the combined potency of multiple elements. This multifaceted collaborative governance approach offers a novel perspective and strategy for optimizing the impact of government sports expenditure on national fitness.

### 6.2 Managerial implications

Initially, it is recommended that the government prioritize the integration of science and technology in sports, alongside the development of sports infrastructure. Additionally, enhancing sports services and promoting widespread dissemination of sports culture should be considered during resource allocation.

Secondly, expenditure on housing security is a crucial factor. This underscores the significance of ensuring a stable living environment for low-income families and constructing basic sports facilities within residential areas to encourage widespread participation in sports. Consequently, when developing housing policies, the government should take into account its potential indirect impact on physical activity participation. It should also strive to increase residents’ awareness of physical activity participation by building community sports facilities.

Ultimately, it is imperative to recognize that no single measure can independently foster an increase in mass physical activity participation levels. Therefore, governments should implement comprehensive strategies in their practical approach. This can be achieved by fostering inter-departmental collaboration, integrating resources, and creating a unified effort to optimize governance effectiveness.

### 6.3 Limitations and prospects

In the analysis of the configuration paths through which government sports expenditure promotes universal sports participation, this study has unveiled the diverse driving factors and their combinatorial effects using the fsQCA approach, offering novel insights for sports policy formulation. While the study has achieved certain results, it is not without limitations. For instance, the sample is confined to 31 provinces (municipalities and autonomous regions) in China, which may restrict the generalizability of the findings. Future research could expand the sample size and include international comparisons to enhance the global applicability of the conclusions. Moreover, the study primarily relies on data from the year 2020, failing to fully capture the long-term dynamic effects of policy impacts. Subsequent studies could employ longitudinal data to provide a more comprehensive assessment of the sustained effects of policies. Currently, fsQCA mainly supports cross-sectional data analysis. In the future, time series or panel data can be processed in combination with other methods. Looking ahead, this study provides an initial dissection of the complex relationship between government sports expenditure and public sports participation, and it is anticipated that future research will build upon these findings to contribute further wisdom to the development of national fitness initiatives.

## Data Availability

The data analyzed in this study is subject to the following licenses/restrictions: the data of sports participation is calculated by the author through searching and collecting relevant data and building indicators. The amount of government financial expenditure is derived from the sports yearbook issued by the General Administration of Sport of the State, but this bibliography is not publicly distributed. If necessary, you can contact the author to provide relevant data sources. Requests to access these datasets should be directed to Wenxin Zhu, wenxinzhu_cumt_edu@126.com.
